# Expressions of Perceived Ageism During Wartime in Israel

**DOI:** 10.1007/s10823-026-09573-0

**Published:** 2026-04-28

**Authors:** Sarit Okun, Assaf Suberry, Liat Ayalon

**Affiliations:** 1 Louis and Gabi Weisfeld School of Social Work, Bar-Ilan University, Ramat Gan, Israel; 2 Department of Community Gerontology, The Max Stern Yezreel Valley College, Afula, Israel

**Keywords:** Age, Older adults, Perceived ageism, TM theory, War, Young adults

## Abstract

Ageism, a widespread social phenomenon, encompasses stereotypes, prejudices, and discrimination based on age. It can be directed towards people of all ages but has received the most attention in relation to older people. While a growing body of research has examined ageism, there remains a notable gap in understanding how age-related dynamics are perceived and interpreted by individuals as ageism during crises in general, and during wartime in particular. This study explores the unique expressions of perceived ageism during “Swords of Iron” war in Israel, using a case study approach. It draws on a survey of 902 Israeli citizens aged 18 to 89 during April 2024. Respondents answered two open-ended questions concerning their general views of ageism during the war as well as their own personal experiences with ageism during the war. In total, 357 (39.6%) of the responses described ageism in the current war, and 156 (17.3%) referred to its presence based on their personal experiences. Six key domains of perceived ageism were identified during the war: reserve military service, employment opportunities, perceptions of mental resilience versus vulnerability, volunteering, opportunities for blood donation, and prioritization in hostage release according to chronological age. These findings demonstrate how crises can intensify the perception of age-based differences and hierarchies, as individuals interpret age-related dynamics through the lens of potential exclusion or bias. This case study highlights the importance of developing intergenerational policies and crisis management strategies that address how age-based practices are experienced and perceived, particularly during extreme conditions such as war.

## Introduction

Ageism, defined as stereotyping, prejudice, and discrimination based on age, affects individuals across the life course (World Health Organization, 2018). It can be directed toward others (“other-directed ageism”) or toward oneself (“self-directed ageism”) (Levy, 2009). Ageism operates on three levels: macro, influencing policies and societal values; meso, affecting institutions such as workplaces and healthcare systems; and micro, shaping individual thoughts, emotions, and behaviors (Ayalon & Tesch-Röömer, 2018). While ageism may carry positive connotations, such as viewing older individuals as experienced and wise or younger individuals as energetic and strong, it is predominantly expressed in negative ways (WHO, 2018). Negative ageism imposes limitations, shapes perceptions of age, and contributes to the internalization of negative stereotypes (Levy, 2009).

Individuals of all ages can experience ageism and be adversely affected by it (WHO, 2018). To date, however, most research has focused on ageism as experienced by older adults across various domains, including healthcare, fashion, law, cinema, and the media (Okun & Ayalon, 2024a). Studies show, for example, that older workers face barriers to employment and promotion (Ng & Feldman, 2012), and that older patients often receive inadequate or dismissive care (Wyman et al., 2018). They are also more likely to experience financial insecurity and negative stereotypical media representation (Gutterman, 2022; Loos & Ivan, 2018; Liddy, 2023). Such expressions of ageism toward older adults have been associated worldwide with reduced health and wellbeing, including a higher risk of mortality (WHO, 2018).

Nonetheless, it is important to distinguish between “age-based institutional criteria,” “perceived ageism,” and “explicit ageism.” Chronological age may be used as a pragmatic basis for policies related to health, safety, or operational capacity, and such criteria do not necessarily reflect prejudice or discriminatory intent (Madva, 2025; Mantel, 2025). For example, age-based restrictions for driving may be based on regulatory, safety, or social considerations rather than bias (Nguyen et al., 2025). In contrast, explicit ageism refers to stereotypes or discriminatory behavior toward individuals based on age (Swift et al., 2019), such as dismissing older adults as incapable or viewing younger individuals as unreliable. Perceived ageism refers to the subjective interpretation of age-related situations as discriminatory, even when they may stem from structural or contextual constraints, such as interpreting exclusion from opportunities or services as unjust age-based treatment (Madva, 2025; Mantel, 2025).

### The Present Study

In recent years, growing attention has been given to ageism during times of crisis, such as pandemics and climate emergencies. The COVID-19 pandemic, for example, underscored the vulnerability of older populations and revealed how ageism can intensify under conditions of uncertainty and threat. Older individuals were frequently portrayed as a burden to society and faced discriminatory treatment in healthcare (Ayalon, 2020), while age-based policies further marginalized older adults. Similarly, in climate change discussions, older adults are often excluded from policymaking, exacerbating their social vulnerability (Ayalon et al., 2022).

However, limited attention has been given to how age-related dynamics are perceived and interpreted during different types of crises, particularly in wartime contexts. The present study, therefore, does not seek to determine whether specific policies or practices implemented during crises constitute explicit ageism, but rather focuses on examining perceived ageism during the “Swords of Iron” war in Israel from the perspective of the public. This war began on October 7, 2023, when Hamas launched a large-scale attack on Israel, resulting in approximately 1,200 fatalities and the abduction of 251 hostages, including children, adults, older individuals, and soldiers (Shrira & Palgi, 2024). During the war, tens of thousands of rockets were fired toward Israel, large-scale civilian displacement occurred, and hundreds of thousands of reservists were mobilized (Gross et al., 2024).

### The Theoretical Ground of the Present Study

Terror Management Theory (TMT) posits that fear of death significantly influences human thoughts and behaviors. To cope with death anxiety, individuals often embrace worldviews that enhance their self-esteem and foster a sense of meaning in their lives (Greenberg & Arndt, 2012). However, in contexts characterized by heightened threat, such as wartime, increased mortality salience may intensify the need for psychological security and reinforce social categorization processes (Oreg & Taubma-Ben-Ari, 2024). These processes may, in turn, shape how individuals interpret social roles, group distinctions, and perceived hierarchies, including those related to age (Oreg & Taubma-Ben-Ari, 2024). Given the scarcity of prior research on perceived ageism in wartime contexts, the present study employs TMT as a conceptual framework for understanding how individuals interpret age-related dynamics during “Swords of Iron” war.

## Methods

The present study employed a mixed method to examine perceptions of ageism during the “Swords of Iron” war in Israel. Data were collected in April 2024 via an online survey administered by a professional polling company (Panel4All). Ethical approval was obtained from the university’s ethics committee (Approval No. 12212/2; December 11, 2023). Participants were provided with a brief definition of ageism (“Ageism represents discrimination, stereotypes, and prejudices toward people of different age groups. Both older and younger people face ageism”) and were asked to respond to two open-ended questions: (1) “Israel is currently at war. In your opinion, how is the phenomenon of ageism manifested during the war?” (2) “Describe any experiences of ageism you personally experienced during the war.” All participants provided informed consent prior to participation and received financial compensation.

### Participants

A total of 902 Israeli adults aged 18 to 89 participated in the study. Participants were recruited using a convenience sampling strategy with stratification by sex and age. Eligibility criteria included being a Hebrew speaking Israeli adult aged 18 years or older with internet access. The inclusion of a wide age range was intended to capture perceptions of ageism across the life course, recognizing that ageism may be experienced and interpreted by both younger and older individuals. To ensure sufficient representation of older adults, individuals aged 65 and above were intentionally oversampled, comprising 26.8% of the sample. The mean age was 47.13 (SD = 18.41), and 55.7% of participants were women. (see Table 1 for detailed sample characteristics).

**Table 1:** Socio-demographic characteristics| *N* = 902

Characteristic	*N*	%
Age group		
18–40	362	40.1
41–64	298	33.0
65–89	242	26.8
Gender		
Woman	502	55.7
Man	400	44.3
Marital status		
Single	203	22.5
Married/partnered	593	65.9
Divorced/widowed	106	11.8
Educational level		
Elementary	10	1.1
High school	234	25.9
Vocation training	204	22.6
Academic	454	50.3
Self-reported health		
Very poor	11	1.2
Poor	28	3.1
Fair	150	16.6
Good	419	46.5
Very good	294	32.6
Economic Status		
Very poor	28	3.1
Poor	85	9.4
Fair	310	34.4
Good	393	43.6
Very good	86	9.5

### Analysis

The analysis proceeded in four stages and combined qualitative thematic analysis with descriptive quantification.

**Stage 1: Classification of responses.** All responses to both open-ended questions were imported into ATLAS.ti (version 8). Each response, defined as a single answer to one open-ended question, served as the primary unit of analysis. Responses were initially classified into two broad categories:

1. responses indicating the presence or intensification of ageism.
2. responses indicating the absence or reduction of ageism.

Irrelevant or non-substantive responses were excluded from the dataset.

As shown in Table 2, regarding the first question, 382 respondents (42.4%) reported that, in their view, ageism was absent or had decreased during the war, whereas 357 respondents (39.6%) reported that it had increased. Regarding the second question, 712 respondents (79.1%) reported no experience of ageism or a decrease in such experiences, while 156 respondents (17.3%) reported an increase in experienced ageism.

**Table 2:** Ageism perceptions and experiences during the war

Survey Questions	Number of Responses	Number Reporting No-Ageism/ Reduced Ageism (%)	Number Reporting increase in ageism (%)
How do you think the phenomenon of ageism manifests during the war?	901	382 (42.4%)	357 (39.6%)
Describe any ageism you experienced during the war.	900	712 (79.1%)	156 (17.3%)

Note: A total of 902 participants took part in the study; however, not all participants responded to both open-ended questions. As a result, the number of responses varies slightly across questions (*n* = 901 and *n* = 900, respectively)

These distributions were examined in a previous study based on the same dataset (Ayalon et al., 2025), which focused on the overall prevalence of ageism and indicated that most respondents did not report experiencing ageism during the war. In contrast, the present study focuses on responses indicating the presence or intensification of ageism in order to examine how such perceptions are constructed and interpreted.

**Stage 2: The corpus.** To enable in depth qualitative analysis, we focused on elaborated responses, defined as responses that included a concrete explanation, example, or justification for the perception of ageism. Across both questions, 390 elaborated responses were identified. These responses could originate from either question, and in some cases participants provided elaborate responses to both questions. This subset constituted the analytic corpus for the thematic analysis.

**Stage 3: Thematic Analysis.** The 390 elaborated responses were analyzed using inductive thematic analysis following Braun and Clarke (2013). The analysis was conducted independently by two members of the research team. The analytic process included:

1. Initial open coding to identify recurring patterns.
2. Grouping codes into indicate themes.
3. Iterative refinement, merging, and exclusion of codes based on conceptual coherence.
4. Final agreement on the thematic structure through discussion with a third researcher.

This process resulted in six central themes representing key domains in which age-related dynamics were interpreted as ageism. The resulting thematic framework, including themes, subcodes, definitions, and illustrative quotes, is presented in Table 3.

**Table 3:** Thematic framework of perceived ageism

Theme	Definition of the Theme	Codes	Example Quotes
1. Perceived Ageism in Reserve Duty	This theme reflects perceptions of age-based exclusion or bias in recruitment to and service within reserve duty during wartime	1. Age-based exclusion from reserve recruitment	1. “I asked to be called up for reserve duty but was told I wasn’t needed” (man, 60)
		2. Perceived loss of societal role among older adults	2. “The older guys in reserve look down on us younger ones” (man, 26)
		3. Age-based tensions within service (younger vs. older)	3. “Younger soldiers made me feel old and outdated” (man, 38)
		4. Stereotypes about competence and authority	
2. Perceived Ageism in Employment	This theme reflects perceived age-based disadvantages in the labor market during wartime	1. Layoffs and exclusion based on age	1. “During the war, I was the first to be pushed aside at work” (woman, 68) 2. “I heard younger employees were put on unpaid leave during the war under downsizing claims, but I assume it was due to their reserve duty” (woman, 25)
		2. Preference for “stable” workers	
		3. Older workers perceivedas expendable	
3. Perceived Ageism in Mental Resilience	This theme reflects perceived age-based disparities in emotional vulnerability, resilience, and access to support during wartime	1. Perceived neglect and social isolation of older adults	1. “It’s a shame that the attention these days goes only to the young” (man, 75)
		2. Perceived devaluation of younger individuals’ need for support	2. “Young people who are not fighting are looked down upon” (woman, 20)
4. Perceived Ageism in Volunteering	This theme reflects perceived age-based exclusion from volunteering opportunities based on assumptions about capability and relevance to the war effort	1. Older adults excluded from contributing	1. “I was told I was too old to contribute” (woman, 67)
		2. Young people perceived as immature/unserious	2. “I didn’t think young people would volunteer seriously” (woman, 24)
		3. Internalized age-based limitations	
		4. Devaluation of willingness to contribute	
5. Perceived Ageism in Blood Donation	This theme reflects expressions of age-based exclusion and stigma in the context of blood donation during wartime	1. Feelings of uselessness	1. “They don’t even let me donate blood” (woman, 71)
		2. Perceived devaluation of contribution	2. “Older people wanted to donate blood during the war but were turned away due to age, which made them feel unnecessary” (man, 45)
6. Perceived Ageism in Hostage Release Prioritization	This theme reflects expressions of age-based prioritization in life-and-death decisions during wartime captivity	1. Youth prioritized as more valuable	1. “Prioritizing children can be seen as ageism” (woman, 23)
		2. Older individuals deprioritized	2. “Older hostages are less valued because they led their lives” (woman, 27)

**Stage 4: Quantification of Themes**. Although the analysis was primarily qualitative, descriptive quantification was used to assess the relative prevalence of themes. Each response could be assigned to one or more themes. Frequencies were calculated based on the number of coded responses per theme, and percentages were derived out of the total 390 coded responses (Table 4). In addition, each coded response was linked to the respondent’s age group (18–40, 41–64, 65–89), enabling comparison of thematic distributions across age groups (Table 5).

**Table 4:** Six key areas of perceived ageism reported during the war

Six Area of Ageism	Number of Reports	Percentage (%)
1. Perceived Ageism in reserve duty service	152	39%
2. Perceived Ageism in employment and work	58	14.9%
3. Perceived Ageism in perceptions of mental resilience	55	14.1%
4. Perceived Ageism in volunteering	51	13.1%
5. Perceived Ageism in blood donation	43	11.1%
6. Perceived Ageism in prioritization for hostage release	31	7.9%
Total (coded responses)	**390**	**100%**

Note: While 123 responses did not include specific examples of how ageism was manifested, 390 responses included explanations regarding the types of ageism that respondents themselves or others experienced during the war

**Table 5:** The distribution of six themes by age group

Age Group	Perceived Ageism in reserve duty service	Perceived Ageism in employment and work	Perceived Ageism in perceptions of mental resilience	Perceived Ageism in volunteering	Perceived Ageism in blood donation	Perceived Ageism in prioritization for hostage release	Total
18–40	70 (49.3%)	30 (21.1%)	20 (14.1%)	12 (8.5%)	2 (1.4%)	8 (5.6%)	142
41–64	50 (42.0%)	20 (16.8%)	18 (15.1%)	16 (13.4%)	6 (5.0%)	9 (7.6%)	119
65–89	32 (24.8%)	8 (6.2%)	17 (13.2%)	23 (17.8%)	35 (27.1%)	14 (10.9%)	129
Total	152 (39.0%)	58 (14.9%)	55 (14.1%)	51 (13.1%)	43 (11.0%)	31 (7.9%)	390

Note: Values represent frequency counts with percentages in parentheses,

## Findings

The present study focuses on responses in which age-related dynamics were perceived and interpreted as ageism during the war. Overall, 357 participants (39.6%) reported that they perceived ageism as present or intensified, and 156 (17.3%) reported personally experiencing ageism during the “Swords of Iron” war (see Table 2). The current analysis is based on 390 elaborated responses that included concrete examples illustrating how ageism was manifested at either the general or personal level.

Analysis of these responses identified six key domains in which perceived ageism was salient during the war in Israel. Importantly, while some of the practices described may be grounded in functional or contextual considerations, participants frequently interpreted them as exclusionary on the basis of age. The six domains are presented in descending order of frequency, such that the first theme represents the most frequently reported domain of perceived ageism. For each theme, we report the number of responses, their relative frequency, and illustrative quotes. Each quote is accompanied by the respondent’s gender and age in parentheses.

### Perceived Ageism in Reserve Duty During Wartime

A total of 152 (39%) of the reports on ageism during the war were related to reserve duty service. Of these, 88 (57.9%) described perceived age-based restrictions on access to reserve duty, while 64 (42.1%) referred to perceived ageism experienced during service.

#### Perceived Ageism in Access to Reserve Duty

88 (57.9%) comments concerning ageism during the war concerned discrimination and exclusion experienced by participants or others due to age restrictions in the reserves. These responses reflected disappointment with the upper age limitations, as many individuals wanted to contribute to the security efforts even beyond the mandatory service age.

52 of the participants referred to the phenomenon in a third person, describing it in general terms or sharing stories of others who faced ageism in this context. For example: “I have a friend who is 39 and was not accepted into his unit because they said he was too old” (man, 46) or “Ageism manifests in the military recruitment process, which is only relevant for a specific age group” (woman, 50). In addition, 36 respondents expressed a personal tone, illustrating the negative feelings they experienced due to not being called for service. For instance: “I asked to be called up for reserve duty but was told I wasn’t needed…” (woman, 60) or:

In the context of reserve duty we are made to feel that there is nothing left for us to contribute to the military at age 70, even though I would love to offer insights and perhaps even practical advice based on my extensive life experience in complex situations (man, 78).

#### Perceived Ageism During Reserve Duty Service

A total of 64 (42.1%) of the reports referred to discrimination and disrespect experienced during reserve service. Approximately half of these responses focused on ageism directed at younger reservists. For example, one participant stated, “The older guys in reserve look down on us younger ones” (man, 26), while another noted, “There’s a sense that older reservists struggle to take orders from younger officers” (woman, 52).

The remaining responses described ageism directed at older reservists. For instance, one participant stated, “In the reserve, younger soldiers made me feel old and outdated” (man, 38), while another noted, “It bothers me that people are surprised when an older person serves in the reserve, as if older individuals are a burden on society” (man, 78).

Overall, this finding suggests that age-related dynamics in reserve duty during wartime operate across both structural and interpersonal levels. While formal age-based eligibility criteria may be grounded in operational and safety considerations, particularly under emergency conditions, they are often experienced and interpreted as exclusionary or as reflecting diminished social value by individuals who wish to contribute.

### Perceived Ageism in Employment During Wartime

A total of 58 (14.9%) of the reports referred to age-related dynamics in employment during the war. This theme appeared across all age groups but was reported more frequently by younger and middle-aged respondents than by older adults.

Participants described employment-related challenges such as layoffs, unpaid leave, and difficulties in securing work, often interpreting these experiences through the lens of age-based disadvantage. For example, one respondent noted, “During the war, there were fewer job openings and more layoffs… both young people and older individuals will struggle more than others to find jobs compared to pre-war times” (man, 37). Another respondent stating that “employers prefer workers without external commitments such as reserve duty or childcare responsibilities, while in some cases retirees are asked to remain due to labor shortages” (man, 75).

Some responses specifically emphasized perceived ageism toward younger workers. For instance, one participant stated, “I think ageism is evident in the fact that young people are put on unpaid leave…” (woman, 25), while another described attempts to dismiss younger employees during workforce reductions (woman, 17). Similarly, younger individuals were described as less stable or reliable in times of crisis (woman, 50). At the same time, other respondents described ageism directed at older workers. For example, one participant noted, “There is discrimination in hiring older workers, especially when combined with military reserve service” (man, 27), while another suggested that older workers are more likely to be dismissed during layoffs (woman, 66).

Overall, this finding suggests that age-related dynamics in the labor market during wartime reflect both structural constraints and interpersonal assumptions. While workforce instability and operational demands may shape employment decisions, these conditions are often interpreted as age-based exclusion by both younger and older workers. At the same time, age-based stereotypes were evident, with younger individuals perceived as unreliable and older individuals as less adaptable or expendable.

### Perceived Ageism in Mental Resilience During Wartime

A total of 55 (10.7%) of the reports referred to age-based perceptions regarding distress and coping during the war. This theme appeared relatively evenly across age groups, suggesting that perceptions of age-related differences in mental resilience were widely shared.

Participants described differing interpretations of how vulnerability and support were distributed across age groups. Some respondents perceived older adults as marginalized and as receiving insufficient physical and mental support. For example, one participant stated, “In my opinion, there is not enough support and assistance for older populations…” (man, 39), while another noted, “It’s a shame that the attention these days goes only to the young” (man, 75). At the same time, other respondents described younger individuals as overlooked or devalued, particularly when they were not directly engaged in combat roles. For example, one participant noted that “young people who are not fighting on the frontlines are looked down upon” (woman, 20), while another suggested that younger individuals may be overlooked in outreach efforts despite also being in distress (woman, 85).

Overall, these findings do not point to a single age group being consistently disadvantaged. Rather, they highlight that similar or parallel experiences may be interpreted as age-based bias by different age groups. This suggests that perceptions of mental resilience during wartime are shaped by competing interpretations of vulnerability and social value, reflecting the multidirectional and interpretive nature of perceived ageism.

### Perceived Ageism in Volunteering During Wartime

A total of 55 (14.1%) reports referred to age-related dynamics experienced by respondents or others when attempting to volunteer. Notably, volunteering efforts during the war involved individuals from a wide range of age groups, suggesting that opportunities for participation were broadly available. Within this context, however, some participants described experiences they interpreted as age-based exclusion.

A subset of responses reflected perceived ageism directed at younger individuals. Thirteen women (aged 18–40) described dismissive attitudes toward their willingness or capability to contribute. For example, one participant noted, “When I volunteered in agriculture, I met people who dismissed me, saying I was too young…” (woman, 24). At the same time, many responses referred to perceived ageism experienced by older adults wishing to volunteer, often emphasizing feelings of exclusion or diminished relevance. Some participants described this in general terms, while others shared personal experiences. For example, one participant noted, “I was told I was too old to contribute, and it frustrated me” (woman, 67).

Overall, these findings do not suggest that a single age group was consistently excluded from volunteering. Rather, they indicate that within broadly inclusive settings, individuals from different age groups may interpret interactions and limitations as reflecting age-based bias. These patterns highlight the multidirectional and interpretive nature of perceived ageism in volunteering contexts during wartime.

### Perceived Ageism in Blood Donation During Wartime

A total of 43 reports (11%) referred to this theme, with many originating from older respondents who are more likely to encounter age-based eligibility criteria. Participants frequently described these restrictions in emotional terms, expressing feelings of uselessness and diminished social value. For example, one respondent stated, “They don’t even let me donate blood!!” (woman, 71), while another noted, “I feel bad and useless when I can’t donate blood because of my age” (man, 75).

Overall, these findings suggest that although age-based criteria for blood donation are typically grounded in medical and safety considerations, they may nevertheless be experienced and interpreted by some participants as exclusionary, particularly in a context were contributing to the collective effort carries heightened symbolic significance.

### Perceived Ageism in Hostage Release Prioritization During Wartime

A total of 31 reports (7.9%) referred to the prioritization of releasing hostages from Hamas captivity, making this the least frequent of the six identified themes. This theme appeared across all age groups but was reported more frequently by older respondents. Notably, despite its relatively low frequency, those who referred to this theme often expressed their views in a particularly assertive and unequivocal manner. For example, one participant stated, “If there is ageism in the war, it is primarily regarding the hostages…” (woman, 28), while another noted, “I don’t think ageism was prominent in the war, except for the release of hostages based on age or health condition” (man, 77).

Participants expressed differing interpretations of prioritization decisions. Some viewed prioritizing younger individuals as devaluing older lives, while others interpreted the prioritization of older or more vulnerable individuals as disadvantaging the young. For instance, one participant noted that prioritizing children “can be seen as ageism” (woman, 23), while another described a “disregard for the lives of older individuals” (woman, 27). At the same time, other responses reflected the opposite perception, suggesting that younger hostages were disadvantaged.

Overall, this final finding highlights that perceptions of age-based prioritization in hostage-release decisions are characterized by competing interpretations, reflecting perceived ageism rather than a consistent view of which age group was disadvantaged.

## Discussion

The study identified six key areas in which perceived ageism was most salient during the “Swords of Iron” war: military service, employment, mental resilience, volunteering, blood donation, and hostage release. Importantly, the practices described in these domains are not necessarily unique to wartime contexts or inherently ageist; rather, the findings highlight how such practices are perceived and interpreted as exclusionary under conditions of crisis. Together, these interpretations point to a broader tendency to experience and frame social practices through age-based lenses during times of heightened threat, with important implications for policy development and intervention strategies.

The first area identified in the survey related to perceived ageism in reserve duty during the war. Both older and younger respondents reported experiencing age-related dynamics as ageist, though in different ways. For older adults, exclusion was often perceived in their rejection from reserve duty, even when they expressed strong willingness to serve and contribute during wartime. While such exclusions may reflect structural or operational considerations, they were frequently experienced diminishing their sense of value and societal role. This aligns with research emphasizing the importance of meaningful social roles for alleviating death anxiety (Kukla et al., 2022). TMT suggests that participating in the war effort may provide individuals with “symbolic immortality” by contributing to a greater cause (Greenberg & Arndt, 2012).

At the same time, the survey also revealed perceived exclusionary experiences among younger participants within the reserve service. These were often attributed to rigid rank hierarchies and military routines (McDermott, 2007). From the perspective of intergenerational conflict theory (North & Fiske, 2012), such dynamics may be interpreted through age-based status hierarchies, whereby older and younger individuals perceive one another through stereotypical lenses. Older participants described being viewed as less capable, whereas younger participants reported feeling undervalued despite their relevant knowledge. These perceptions point to how intergenerational dynamics may be experienced as age-based inequality, highlighting the importance of addressing both structural arrangements and age-related assumptions in understanding perceived ageism in this context.

The job market emerged as the second significant area in which age-related dynamics were perceived as reflecting ageism during the war. Participants described employment-related challenges affecting both younger and older workers, often interpreting these experiences through the lens of age-based exclusion. While labor market conditions during wartime are shaped by structural constraints, such as workforce instability, reserve duty obligations, and concerns about availability, these conditions were frequently experienced as age-related disadvantages. Existing literature suggests that employment practices often rely on age-based assumptions regarding adaptability, productivity, and stability (Kite et al., 2005; Henkens et al., 2005; Finkelstein et al., 2013; Posthuma & Campion, 2007), which may inform how individuals interpret workplace dynamics during crises. From this perspective, younger participants described being perceived as unreliable or inexperienced, whereas older participants reported being viewed as less adaptable or expendable.

Mental resilience emerged as a third area in which age-related dynamics were perceived as reflecting ageism during the war. Participants described a perceived tendency to prioritize younger individuals in the allocation of mental health support, while older adults were often experienced as receiving less attention. While such patterns may reflect broader assumptions about vulnerability and need during crises, they were frequently interpreted as age-based bias. This is particularly salient given the substantial impact of the conflict on older populations, including high rates of casualties and displacement (Okun & Ayalon, 2024b).

These perceptions may be partly explained by the societal assumption that younger individuals possess greater psychological resilience, which can function as a defense mechanism reinforcing a sense of invulnerability under existential threat (Ben-Simon & Konstantinov, 2024). Such beliefs resonate with ageist stereotypes that associate youth with vitality and resilience while framing old age as decline (Binyamin & Brender-Ilan, 2023), thereby shaping how age-related differences in support are interpreted as ageism across different age groups.

Volunteering during wartime represents a fourth area in which age-related dynamics were perceived as reflecting ageism. Participants, particularly older adults, described barriers to participation despite their willingness to contribute, often interpreting these experiences as exclusions based on age. While such barriers may reflect structural or practical considerations, they were frequently experienced as limiting opportunities for meaningful engagement. This is notable given that volunteering is often viewed as a pathway that enables older adults to remain active despite structural barriers to paid employment (Lam et al., 2023), and as a source of purpose and social connection that can alleviate death anxiety (Lam et al., 2023; Sundström et al., 2021). From this perspective, perceived exclusion from volunteering opportunities may undermine individuals’ sense of agency and reinforce interpretations that their contributions are less valued. These findings highlight the importance of fostering inclusive volunteer frameworks that are attentive not only to participation, but also to how opportunities are experienced across age groups (Warburton & McLaughlin, 2005).

The fifth area in which age-related dynamics were perceived as reflecting ageism concerned restrictions on blood donation during the war, with blood donation reflecting a gesture symbolizing life and continuity (Romero-Domínguez et al., 2021). Participants, particularly older adults, described age-based limitations on donation, often interpreting these restrictions as exclusionary. While such criteria may be grounded in medical or safety considerations (Magen David Adom, 2023), they were frequently experienced as diminishing their perceived social value. From a TMT perspective, situations involving heightened mortality salience may increase the symbolic significance of contributing to collective survival (Greenberg & Arndt, 2012). In this context, being unable to donate blood may be interpreted as a form of marginalization, particularly when younger individuals are perceived as more vital to society’s future.

The sixth area in which age-related dynamics were perceived as reflecting ageism concerned hostage release decisions during the war. Although relatively infrequent, this theme appeared across all age groups and was reported most often by older respondents. Participants interpreted these decisions through the lens of age-based value and vulnerability, with competing perceptions regarding which group was disadvantaged. These divergent interpretations may reflect heightened mortality salience during wartime. According to TMT, awareness of death intensifies reliance on culturally embedded frameworks of meaning and value (Greenberg & Arndt, 2012). Accordingly, age-based prioritization may be understood through culturally grounded meanings associated with life stage and social value, highlighting that such decisions are not experienced uniformly but are interpreted in multiple and sometimes conflicting ways.

## Conclusion

“Swords of Iron” war serves as a unique case for understanding how wartime conditions intensify the perception of ageism, as age-related dynamics are interpreted through the lens of bias under conditions of existential threat. From a TMT perspective, heightened mortality salience increases reliance on age-based categories, shaping how individuals construct differences in roles, vulnerability, and social value (Greenberg & Arndt, 2012; Oreg & Taubman-Ben-Ari, 2024). These processes may simultaneously generate perceptions that younger individuals are favored as stronger or more vital, while older adults are viewed as more vulnerable, alongside counter-perceptions that younger individuals are less experienced or less essential contributors.

Importantly, the current findings advance TMT-based research in the Israeli context (Oreg & Taubman-Ben-Ari, 2024) by demonstrating that mortality salience is not only expressed through symbolic and cultural mechanisms but also operates at the level of everyday social interpretation. Specifically, the findings show how existential threat shapes the perception of age-based distinctions as discriminatory across multiple domains of daily life. By moving beyond a focus on symbolic processes, the present study highlights the translation of TMT mechanisms into lived social experiences of age and ageism during wartime.

These insights carry important implications for policy and practice. Crisis management strategies should address not only the structural use of age-based criteria, but also how such practices are perceived and interpreted across age groups. Given that the findings reflect subjective perceptions rather than direct evidence of institutional discrimination, addressing perceived exclusion becomes a critical component of inclusive policy design. A multigenerational approach may therefore play a key role in mitigating perceived age-based divisions and fostering intergenerational solidarity under conditions of crisis.

### Limitations and Future Research

This study has several limitations that should be acknowledged. First, the sample was based on convenience sampling and therefore cannot be considered nationally representative. Participants were recruited through an online survey, which may have favored individuals with internet access, digital literacy, and availability to respond under wartime conditions, while underrepresenting groups such as evacuees, reservists, and others facing acute disruption. Accordingly, the findings should be interpreted as reflecting the views of an accessible segment of the population rather than those of the broader Israeli public.

Second, the study relied on only two open-ended questions. Although these questions yielded valuable insights into perceptions of ageism during wartime, they necessarily limited the depth, nuance, and contextual detail that could be captured. More extensive qualitative methods, such as in-depth interviews, focus groups, or mixed-method designs, could provide a fuller understanding of how age-related experiences are interpreted and negotiated during crises.

Third, the study was conducted within the highly specific cultural and national context of Israeli society during wartime. Perceptions of ageism are likely shaped by local social norms, intergenerational relations, security related realities, and the broader sociopolitical context. Caution is therefore needed in generalizing these findings to other societies or emergency settings. Studies in other cultural and national contexts are needed to clarify which patterns are context specific and which may be more universal.

## Data Availability

Data, analytic methods or materials will be available to other researchers for replication purposes via our website: [https://www.no2ageism.com](https:/www.no2ageism.com).
